# Infliximab as an alternative therapy for refractory adult onset Kawasaki disease: A case report

**DOI:** 10.1097/MD.0000000000012720

**Published:** 2018-10-05

**Authors:** Takeshi Kawaguchi, Yuki Rikitake, Toshihiro Tsuruda, Chihiro Kawata, Mao Rikitake, Kosho Iwao, Ayako Aizawa, Yumi Kariya, Motohiro Matsuda, Syunichi Miyauchi, Kunihiko Umekita, Ichiro Takajo, Akihiko Okayama

**Affiliations:** a Department of Rheumatology, Infectious Diseases and Laboratory Medicine; b Department of Circulatory and Body Fluid Regulation, Faculty of Medicine, University of Miyazaki, Miyazaki, Japan.

**Keywords:** adult-onset Kawasaki disease, coronary artery aneurysm, high-dose intravenous immunoglobulin, infliximab, vasculitis

## Abstract

**Rationale:**

Kawasaki disease (KD) is an acute febrile illness predominantly affecting children less than 5 years of age and characterized by systemic inflammation in all medium-sized arteries. Adult-onset KD (AKD) is rare with only 105 case reports published. Recently, the efficacy of infliximab (IFX) for patients with refractory KD has been demonstrated.

**Patient concerns:**

A previously healthy 24-year-old man was admitted because of a persistent fever, and elevated serum level of AST, ALT, LDH, and CRP.

**Diagnosis:**

The patients met the diagnostic criteria for KD based on the findings of persistent fever, polymorphous exanthema, unilateral cervical lymphadenopathy, non-purulent palpebral conjunctivitis and membranous desquamation. Echocardiogram revealed the dilatation at the proximal sites of the right coronary artery (7.9 mm) and left anterior descending artery (5 mm). The patient was treated with high-dose intravenous immunoglobulin (1 g/kg/day for 2 days) and ASA (100 mg daily). However, his fever and arthralgia persisted.

**Interventions:**

He was administered single 5 mg/kg doses of IFX.

**Outcomes:**

He became afebrile the next day and his arthralgia improved.

**Lessons:**

We report the first case of administration of IFX in a patient with AKD refractory to intravenous immunoglobulin (IVIG), and successful reduction of systemic inflammation. However, the effectiveness of IFX in the regression of coronary artery aneurysm remains to be determined.

## 1 Introduction

Kawasaki disease (KD), or mucocutaneous lymph node syndrome, is an acute febrile illness that predominantly affects children less than 5 years of age. KD is characterized by systemic inflammation in all medium-sized arteries. Kawasaki first described the clinical presentation in Japan in 1967,^[1]^ and Kato described its cardiac complications.^[2]^ The cause of KD remains unknown. Rowley suggested that infection with a novel RNA virus in the respiratory tract may be associated with its etiology.^[3]^ In children, administration of high-dose intravenous immunoglobulin (IVIG) and acetylsalicylic acid (ASA) during the acute phase of KD reduced the frequency of coronary complications.^[4]^ Recently, some reports have demonstrated the efficacy of infliximab (IFX) for patients with refractory KD.^[5,6]^

Adult-onset KD (AKD) is rare, and it is difficult to make a diagnosis of AKD in the acute phase. Our investigation of the literature yielded only 105 published case reports.^[7–13]^ Moreover, there have been no case reports describing AKD treated with IFX to date. In the present report, we describe a case of AKD which was refractory to IVIG, but which successfully reduced systemic inflammation with IFX.

## 2 Case presentation

A previously healthy 24-year-old man presented with fever, diarrhea, and erythema of his lower limbs. He was admitted to the local hospital. Blood tests showed an elevated serum levels of aspartate transaminase (AST), alanine transaminase (ALT), lactate dehydrogenase (LDH), and C-reactive protein (CRP). Rickettsial infection was suspected. Administration of intravenous antibiotics (levofloxacin and minomycin) failed to yield an improvement.

**Figure 1:**
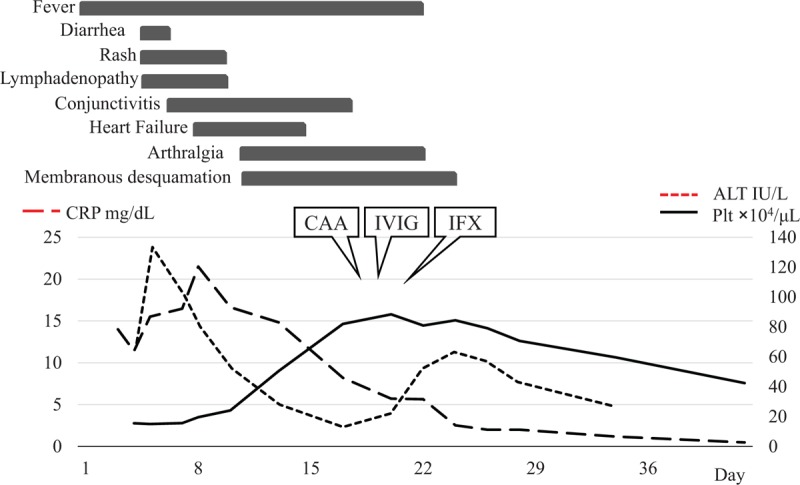
Clinical course of this patient. ALT = alanine transaminase, CAA = coronary artery aneurysms, CRP = C-reactive protein, IFX = infliximab, IVIG = intravenous immunoglobulin, Plt = platelet.

**Figure 2:**
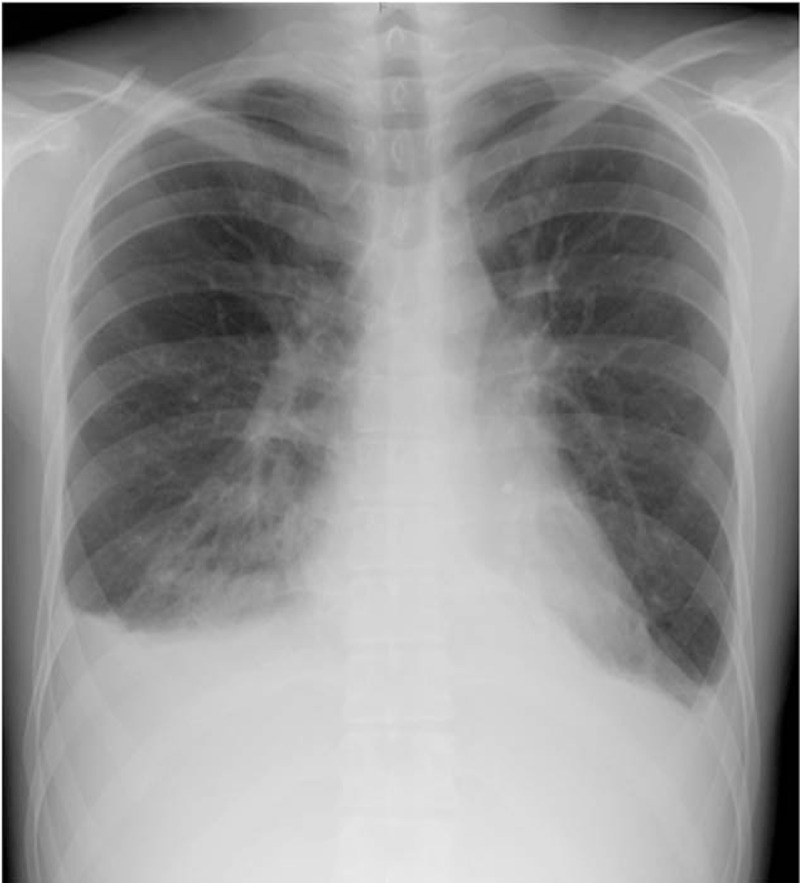
Chest X-ray performed on day 10 demonstrated the dilatation of chest-thoracic ratio and bilateral pleural effusion.

**Figure 3:**
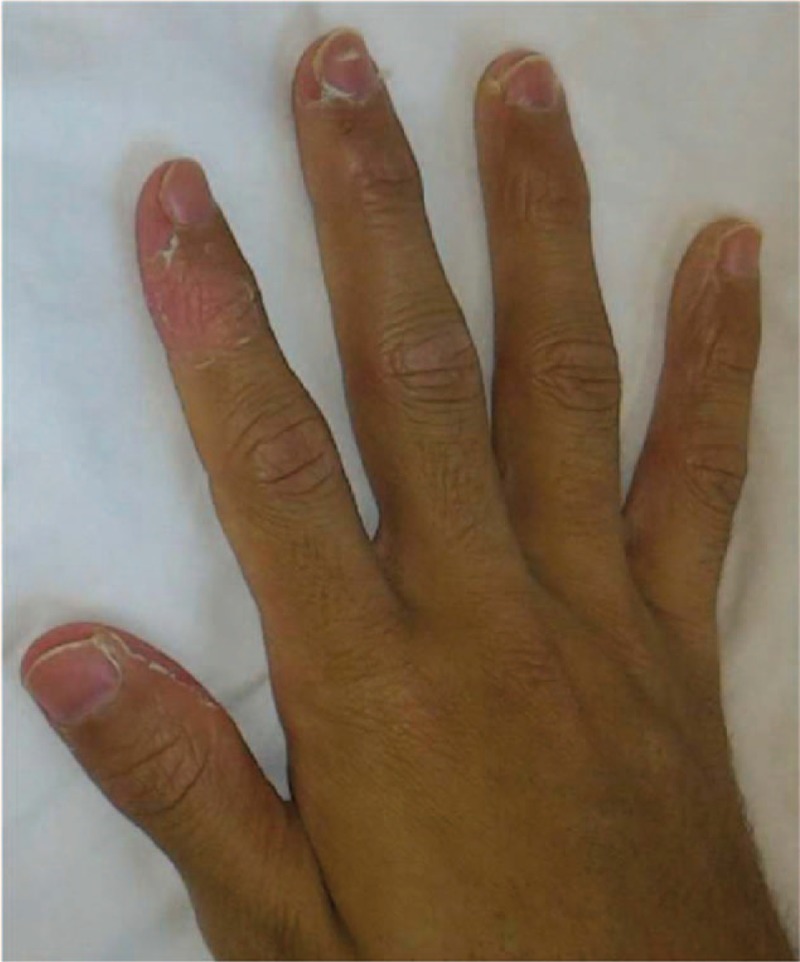
The patient's fingertips exhibited membranous desquamation on day 11.

On day 5, the patient was transferred to our hospital complaining of hypochondralgia and right lower abdominal pain. Physical examination revealed a temperature of 39 °C, non-purulent palpebral conjunctivitis, left unilateral cervical lymphadenopathy, and rash on his lower limbs (Fig. 1). Laboratory tests showed leucocytosis with a predominance of polymorphonuclear leucocytes, and increased serum levels of AST, ALT, LDH, and CRP (Table 1). Leptospirosis and *Yersinia pseudotuberculosis* infection were suspected initially. He was treated with intravenous antibiotics (Cefoperazone/Sulbactam) on day 7. Despite this treatment, his temperature remained at the level of 39 °C. Blood bacterial cultures were negative. Serologic tests showed no significant results for *Rickettsia*, *Leptospira*, adenovirus, hepatitis A virus, hepatitis B virus, hepatitis C virus, cytomegalovirus, Epstein–Barr virus, human immunodeficiency virus, and parvovirus B19. On day 10, he complained of shortness of breath, and chest X-ray showed a 52% chest-thoracic ratio and bilateral pulmonary congestion (Fig. 2). Electrocardiogram showed widespread ST elevations at II, III, aV_F_, and V_2-6_ leads. Transthoracic echocardiogram revealed decreased diffuse left ventricular wall motion and pericardial effusion. Troponin T and BNP levels were elevated at 0.08 ng/mL (normal range <0.02 ng/mL) and 1092.6 pg/mL (normal range <18.4 pg/mL), respectively. On day 11, he complained of membranous desquamation of the fingertips (Fig. 3) and arthralgia of shoulders and wrist. Subsequently, on day 17, echocardiogram revealed dilatation at the proximal sites of the right coronary artery (7.9 mm) and left anterior descending artery (5 mm). The patient met the diagnostic criteria for KD except for changes in lips and oral cavity, and he was diagnosed with AKD. Although cardiac dysfunction, diarrhea, rash, lymphadenopathy, and conjunctivitis improved gradually, fever and arthralgia continued.

**Table 1:**
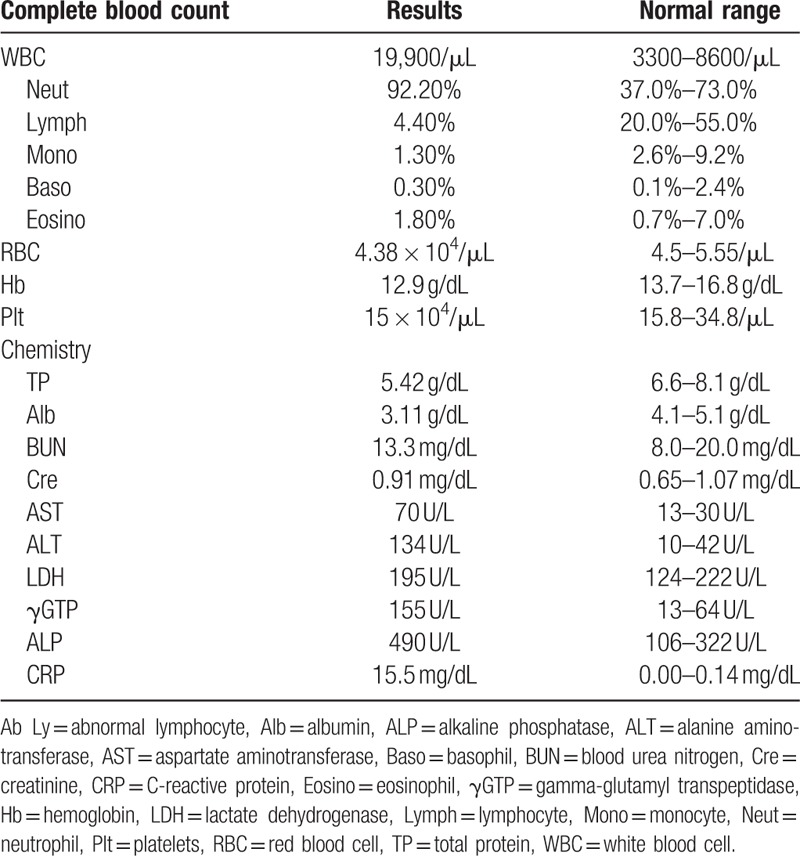
Laboratory data on the admission of our hospital.

On day 18 to 19, the patient was treated with high-dose IVIG (1 g/kg/day for 2 days) and ASA (100 mg daily). However, his fever and arthralgia persisted. Therefore, on day 21, he was treated with IFX (5 mg/kg given as a single intravenous infusion). He became afebrile the next day and his arthralgia improved. The levels of ALT and CRP peaked within the first 10 days; however, the level of ALT increased temporarily after the treatment with IVIG and IFX. Platelet count was highest in the subacute phase (day 20) after IVIG (Fig. 1). On day 30, he was discharged without symptoms. He was followed as an outpatient on a low dose of ASA.

**Figure 4:**
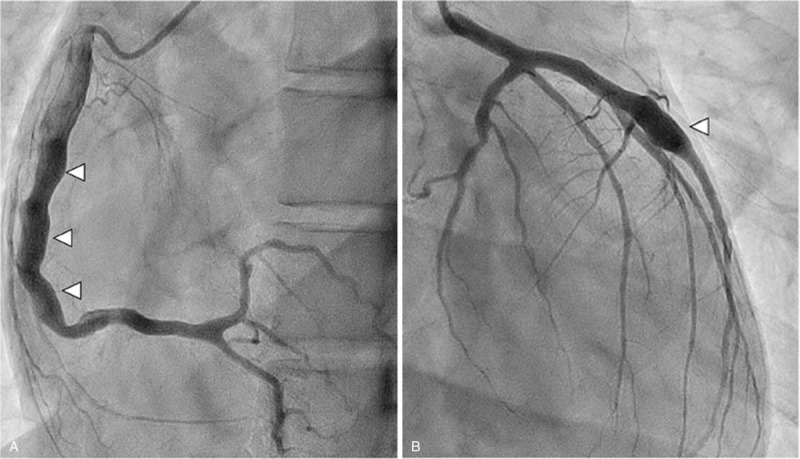
Coronary angiography performed 6 months later demonstrated dilatation/aneurysm at segment 1 to 3 of the right coronary artery (shown by arrows) (A) and segment 7 of the left anterior descending artery (shown by arrow) (B) without stenosis.

Six months later, coronary angiography was performed to determine the exact extent of coronary artery aneurysm, stenosis, and thrombosis. This revealed dilatation at segments 1 to 3 (6.5 mm) of the right coronary artery and a saccular aneurysm at segment 7 (7.0 mm) of the left anterior descending artery (Fig. 4A and B) without any stenosis and thrombosis.

## 3 Discussion

The diagnosis of KD is based on the principal clinical findings of fever persisting a minimum of 5 days and the presence of at least 4 of the following 5 features: changes in extremities that initiate with erythema and edema and progress to desquamation of fingertips, polymorphous exanthema, conjunctivitis, changes in lips and oral cavity, and cervical lymphadenopathy.^[14]^ Since the first report of KD in Japan, many patients in childhood have been reported worldwide in 2007 to 2008. The annual incidence in Japan was 216.9 per 100,000 children younger than 5 years of age.^[15]^ On the other hand, the incidence of KD occurred in adults is very rare with only 106 cases, including ours, reported in the literature.^[7–13]^ Gormard-Mennesson et al reviewed the clinical characteristics of 81 cases of AKD.^[16]^ Average age was 29 years old. Fifty-four patients (67%) were male. Sève et al. summarized the clinical characteristics of 57 cases of AKD.^[17]^ Cervical lymphadenopathy (93% of adults vs 75% of children), hepatitis (65% of adults vs 10% of children), and arthralgia (61% of adults vs 24% of children) were more frequently found in adults than in children. Thrombocytosis (56% of adults vs 100% of children) and coronary aneurysms (5% of adults vs 20% of children) were less common in adults. Our case demonstrated congestive heart failure due to acute myocarditis, and there have been case reports describing AKD complicated by heart failure.^[18,19]^ Approximately 5% of children with KD in the continental United States present with cardiovascular collapse and hypotension requiring volume expanders, vasoactive agents, or transfer to intensive care.^[20–22]^

It is important to note that diagnosis of KD in Adults (n = 50/70 cases, 71%) requires more than 10 days from onset^[16]^ due to its rareness in adults and the lack of specific diagnostic test for AKD. In children, 70% of KD patients received IVIG treatment within 5 days from onset, and only 1.5% of KD patients needed 10 or more days to receive IVIG treatment.^[23]^ Similar to our case, the majority of patients (n = 44/57, 77%) were treated initially with antibiotic agents.^[17]^ Our patient had persistent fever and met 4 of the 5 features except for changes in lips and oral cavity. AKD should be included in the differential diagnosis when a patient presents with unexplained fever, mucocutaneous changes, lymphadenopathy, and congestive heart failure.

Laboratory tests may be useful in supporting a diagnosis of KD, although most parameters are nonspecific. Consistent with our cases, Tremoulet et al^[24]^ reported that white blood cell count, erythrocyte sedimentation rate, and CRP values were highest and hemoglobin was lowest in the acute phase before IVIG administration; however, platelet count was highest in the subacute phase after the treatment.

The consensus recommendation for the treatment of KD in children is high-dose IVIG (2 g/kg given as a single intravenous infusion) and moderate- (30–50 mg/kg/day) to high-dose (80–100 mg/kg/day) of ASA.^[20]^ Because of the potentially severe outcome of coronary artery complications, IVIG should be administered within 10 days of onset or as soon as possible after diagnosis. In adults, the management of KD has not been established. Sève et al^[17]^ summarized case reports on the treatment and evolution of AKD. Twenty-one cases were treated with low-dose aspirin and 18 cases were treated with IVIG (0.4 g/kg/day for 5 days, or 2 g/kg for 1 day). Both treatments were favorable in the majority of cases.

Approximately 10% to 20% of children with KD revealed persistent or recurrent fever after primary therapy with IVIG plus ASA.^[25]^ Treatment options for IVIG-resistant cases include IVIG with prednisolone, or IFX.^[20]^ The evidence of IVIG with prednisolone is limited. Kobayashi et al^[26]^ reported a retrospective study which showed the efficacy of IVIG with prednisolone using a database of 359 consecutive IVIG-resistant patients. On the other hand, some reports have demonstrated the efficacy of IFX for patients with refractory KD. Weiss et al^[27]^ first reported IFX administration to a 3-year-old patient with KD unresponsive to multiple doses of IVIG and methylpredonisolone. The efficacy and safety of IFX treatment in IVIG-resistant patients with KD has been proven^[28]^ in a report by Burns et al of IFX (5 mg/kg intravenously over 2 hours) versus a 2nd infusion of IVIG for patients who had fever at least 36 hours after the end of initial IVIG infusion. IFX infusion led to a cessation of fever within 24 hours in 11 of 12 subjects. Masuda et al^[6]^ analyzed 434 patients with KD who received IFX between 2005 and 2014. In their report, IFX was administered as an additional treatment. After IFX, 84% of KD patients (n = 363/434) became afebrile within 2 days, although 27% of patients (n = 119/434) received additional treatment. Mori et al^[29]^ reported efficacy and safety in a phase 3 trial of IFX for Japanese pediatric patients with KD showing persistent fever after initial IVIG. Reduction of body temperature to below 37.5 °C within 48 hours was achieved with greater frequency with IFX (76.7%) than with IVIG (37.0%).

The safety profile of IFX in adult patients with KD is unknown. According to postmarketing surveillance of the safety profile of IFX in Japanese patients with rheumatoid arthritis, the incidence rates of total and serious adverse events (AEs) were reported to be 28% and 6.2%, respectively.^[30]^ Serious infusion reactions were observed in 0.5%. Infections including bacterial pneumonia (2.2%), tuberculosis (0.3%), and *Pneumocystis jiroveci* pneumonia (0.4%) were observed. It is necessary to monitor vital signs at the beginning of IFX and to check patient condition, especially respiratory disorders, for several months after treatment with IFX. In our case, the serum level of ALT was temporarily increased after administration of IVIG and IFX (Fig. 1). Because the frequency of hepatobiliary disorder with IFX in patients with RA was low,^[30]^ we speculated that IVIG was the cause.

Our case required 17 days for a diagnosis of AKD with complicating coronary artery aneurysms (CAA), and we decided to treat the patient with IFX following IVIG administration. As far as we know, this is the first case in which IFX was administered following IVIG in an adult patient with KD. Our case exhibited no fever following the first infusion of IFX, and CRP level and platelet count decreased thereafter. The effectiveness of the IFX administration on CAA remission remains unknown because coronary angiography performed 6 months after treatment with IFX revealed CAA equivalent in size to that observed at the acute phase assessed by echocardiogram. The effectiveness of IFX therapy on the development of CAA remains inconclusive. Previous studies have reported the statistically insignificant size of coronary artery aneurysm or the relatively low appearance of ectasia in IFX in addition to IVIG versus IVIG treatment.^[29,31]^

We only reported 1 adult case of KD treated with IFX refractory to IVIG, and so cannot make a conclusion about the safety and efficacy of IFX therapy to potential AKD patients. Specifically, long-term follow-up is necessary to determine whether IFX therapy can decrease the size of coronary artery aneurysm. Future studies on AKD patients treated with IFX in addition to IVIG are needed.

In conclusion, we reported the first case of the administration of IFX refractory to IVIG in a patient with AKD resulting in successful reduction of systemic inflammation. We learned in the present case that KD should be included in the differential diagnosis even if an adult patient presents with unexplained fever, mucocutaneous changes, lymphadenopathy, and congestive heart failure. In addition, the present case suggests that IFX can be an effective alternative therapy for refractory AKD.

## Acknowledgments

The authors thank Drs. Kyouhei Kondou and Masako Harada, Division of Pediatrics, Faculty of Medicine, University of Miyazaki, and Dr. Keigo Nakatani, Department of Pediatrics, Miyazaki Prefectural Miyazaki Hospital for their support for the diagnosis and treatment of the patient.

## Author contributions

**Data curation:** Takeshi Kawaguchi, Yuki Rikitake, Chihiro Kawata, Mao Rikitake, Kosho Iwao, Ayako Aizawa, Yumi Kariya, Motohiro Matsuda, Shunichi Miyauchi, Ichiro Takajo.

**Project administration:** Takeshi Kawaguchi.

**Supervision:** Toshihiro Tsuruda.

**Visualization:** Takeshi Kawaguchi.

**Writing – original draft:** Takeshi Kawaguchi.

**Writing – review & editing:** Takeshi Kawaguchi, Toshihiro Tsuruda, Kunihiko Umekita, Akihiko Okayama.
